# Three-dimensional visualization of the total mesorectal excision plane for dissection in rectal cancer surgery and its ability to predict surgical difficulty

**DOI:** 10.1038/s41598-023-29426-x

**Published:** 2023-02-06

**Authors:** Yuzo Nagai, Kazushige Kawai, Hiroaki Nozawa, Kazuhito Sasaki, Koji Murono, Shigenobu Emoto, Yuichiro Yokoyama, Hiroyuki Matsuzaki, Shinya Abe, Hirofumi Sonoda, Yuichiro Yoshioka, Takahide Shinagawa, Soichiro Ishihara

**Affiliations:** Department of Surgical Oncology, Faculty of Medicine, The University of Tokyo, 7-3-1 Hongo, Bunkyo-ku, Tokyo, 113-0033 Japan

**Keywords:** Cancer, Gastrointestinal system, Predictive markers

## Abstract

Total mesorectal excision (TME) for rectal cancer is often technically challenging. We aimed to develop a method for three-dimensional (3D) visualization of the TME dissection plane and to evaluate its ability to predict surgical difficulty. Sixty-six patients with lower rectal cancer who underwent robot-assisted surgery were retrospectively analyzed. A 3D TME dissection plane image for each case was reconstructed using Ziostation2. Subsequently, a novel index that reflects accessibility to the deep pelvis during TME, namely, the *TME difficulty index*, was defined and measured. Representative bony pelvimetry parameters and clinicopathological factors were also analyzed. The operative time for TME was used as an indicator of surgical difficulty. Univariate regression analysis revealed that sex, body mass index, mesorectal fat area, and TME difficulty index were associated with the operative time for TME, whereas bony pelvimetry parameters were not. Multivariate regression analysis found that TME difficulty index (*β* = − 0.398, *P* = 0.0025) and mesorectal fat area (*β* = 0.223, *P* = 0.045) had significant predictability for the operative time for TME. Compared with conventional bony pelvimetry parameters, the TME difficulty index and mesorectal fat area might be more useful in predicting the difficulty of rectal cancer surgery.

## Introduction

Colorectal cancer is a serious public health problem. It has the third highest incidence rate of all types of cancer and is the second leading cause of cancer-related deaths worldwide^1^. Particularly, improving the treatment outcome of lower rectal cancer is of considerable importance owing to its high incidence of local recurrence and the high potential for distant metastasis after surgical resection.

Total mesorectal excision (TME) was first reported in 1982 by Heald^2^. It is currently the gold standard for the surgical treatment of rectal cancer. En bloc resection of the mesorectum by sharp dissection is critical to reduce the incidence of local recurrence and improve the survival outcome^3,4^. In recent years, TME via minimally invasive approaches, such as laparoscopic surgery and robotic surgery, has been widely accepted with feasible short-term results and oncological safety^5–7^. Transanal TME (TaTME) is also widely used and provides excellent visualization of the distal rectum^8^. However, laparoscopic or robotic TME is still often challenging because of the technical difficulties caused by various clinical and anatomical factors. For example, a narrow pelvic space or bulky mesorectum could limit the operability of the laparoscopic forceps or robotic arms, which may result in such serious postoperative morbidity as anastomotic leakage or in compromised oncological curability due to an incomplete TME^9^.

Previous studies have demonstrated the usefulness of pelvimetry measures by computed tomography (CT) or magnetic resonance imaging (MRI) for predicting the difficulty of rectal cancer surgery^9–20^. For example, a recent systemic review reported that a shorter interspinous distance, a shorter intertubercle distance, a smaller pelvic inlet, and a greater pubic tubercle height were associated with increased surgical difficulty in at least one or more studies^21^. However, these bony pelvic indicators were not measured based on the actual TME dissection plane. Therefore, we assumed that the ability of conventional bone-based indicators to predict the difficulty of rectal cancer surgery would be limited and that an indicator based on the TME dissection plane would be more suitable.

In this study, we first developed a method to visualize the TME dissection plane from preoperative CT and MRI images with a workstation software. Using the created three-dimensional (3D) images of the TME dissection plane, we then defined and measured a new index, namely, the *TME difficulty index*. The usefulness of the TME difficulty index for predicting the difficulty of TME in lower rectal cancer patients was retrospectively evaluated along with the conventional pelvimetry indicators and clinical factors.

## Methods

### Patients

Sixty-six consecutive patients with lower rectal cancer who underwent robot-assisted surgery between 2018 and 2021 at our institution were analyzed. Their clinical, surgical, and pathological data were collected from medical records. All cancers were located in the lower rectum, below the peritoneal reflection. The distance from the anal verge to the lower border of the tumor was measured by colonoscopy or digital examination. Preoperative chemoradiotherapy (CRT) with long-course radiation and 5-fluorouracil-based chemotherapy was indicated for patients with clinical T3 or higher tumors. All patients underwent chest and abdominopelvic contrast-enhanced CT and pelvic MRI preoperatively for cancer staging. The cancer stage was identified according to the *TNM Classification of Malignant Tumors, 8th Edition*, of the Union for International Cancer Control^22^. Radial margin (RM) was defined as the closest distance between the tumor tissue and the lateral resection margin. The distal margin (DM) was defined as the distance between the lower verge of the primary tumor (or scar tissue in patients with a pathological complete response) and the distal verge of the bowel specimen.

All robotic surgeries were performed by qualified surgeons under the Endoscopic Surgical Skill Qualification System in Japan^23^. The da Vinci Xi System (Intuitive Surgical, Sunnyvale, CA, USA) was used in all cases. Regardless of the type of surgery (i.e., low anterior resection [LAR], intersphincteric resection [ISR], or abdominoperineal resection [APR]), transabdominal TME dissection to the level of the anorectal junction was conducted as the standard procedure in all patients. Lateral pelvic lymph node dissection was performed in patients with clinically suspected metastasis. A preventive diverting stoma was created when indicated.

Patients with inappropriate preoperative CT or MRI images and female patients with large uterine fibroids were excluded from the study. This study was performed in line with the principle of the Declaration of Helsinki and was approved by the local ethics committee of the University of Tokyo Hospital under approval no. 3252-(13). Written informed consent for data use was obtained from all the patients.

### Creation of 3D images of the TME dissection plane

**Figure 1:**
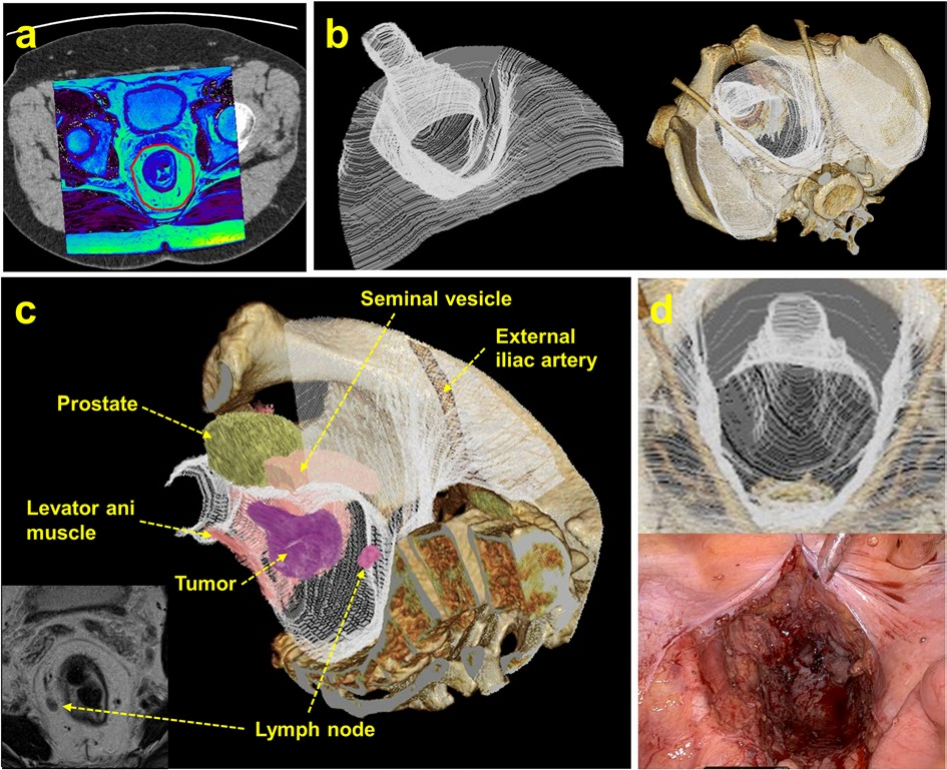
Three-dimensional (3D) visualization of the total mesorectal excision (TME) dissection plane. (**a**) A T2-weighted magnetic resonance image was superimposed on the contrast-enhanced computed tomography image. The line of TME dissection was manually traced (red line). (**b**) Created 3D images of the TME dissection plane. (**c**) A 3D image of the TME dissection plane in a male patient. The primary tumor, swollen lymph node, pelvic bone, external iliac artery, levator ani muscle, prostate, and seminal vesicle are also shown. (**d**) Comparison of the 3D TME dissection plane image and intraoperative image of the pelvic cavity after TME and resection of the rectum in the same patient.

The creation of 3D images of the TME dissection plane was performed using Ziostation2 (Ziosoft, Tokyo, Japan). Ziostation2 is a commercially available workstation software capable of producing highly precise 3D images of various target organs and vessels from CT and MRI images. As shown in Fig. 1a, the pelvic T2-weighted axial plane of the MRI was first superimposed on the contrast-enhanced axial plane of the CT in the same patient; then, the line of TME dissection was manually traced on the MRI and CT images. Subsequently, a 3D image of the TME dissection plane was reconstructed by repeating this operation in the cranial–caudal direction and combining the acquired images (Fig. 1b). In Fig. 1c, the 3D TME dissection plane is depicted along with other structures, such as the pelvic bone, common and external iliac arteries, levator ani muscle, prostate, and seminal vesicle in male patients. This protocol also allowed for the location of the primary tumor and swollen lymph nodes within the mesorectum to be determined. Figure 1d, compares a 3D TME dissection plane image with an actual intraoperative pelvic image after completion of the TME and transection of the rectum in the same patient.

### Definition and calculation of the TME difficulty index

**Figure 2:**
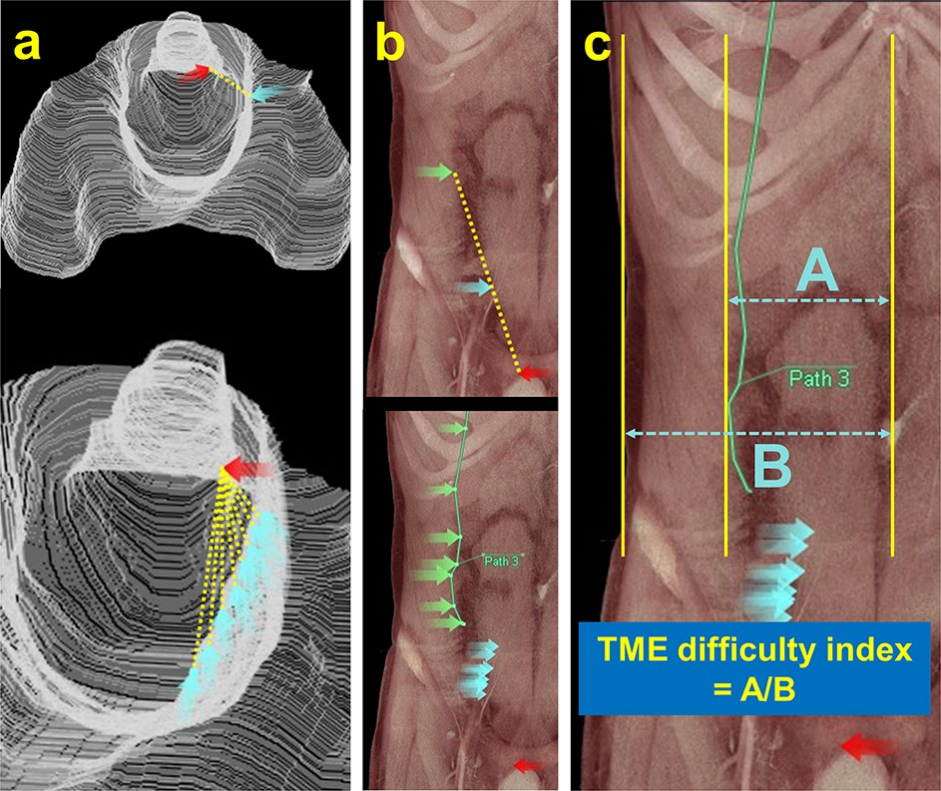
Definition and calculation of the total mesorectal excision (TME) difficulty index. (**a**) The right side of the anal canal at the level of the anorectal junction was marked on the three-dimensional TME dissection plane (red arrow). Subsequently, the points from where the marked point on the right side of the anal canal could be accessed without any hindrance were marked (blue arrows). (**b**) By extrapolation of the line connecting the red arrow and blue arrows to the abdominal surface (green arrow), the right boundary on the abdominal surface from where the marked point on the right side of the anal canal can be accessed without any hindrance was determined (green line). (**c**) The maximum distance from the navel to the right boundary (A) and the distance from the navel to the right edge of the abdominal wall (B) were measured. The TME difficulty index was defined as the ratio of these two distances (A/B) on the assumption that the smaller the index, the greater the difficulty of accessing the deep pelvis and, consequently, the greater the difficulty of TME.

To develop an indicator for predicting the difficulty of TME based on the created TME dissection plane images, we focused on the right side of the anal canal, which is one of the most difficult points to access during laparoscopic or robotic TME. The right side of the anal canal at the level of the anorectal junction was marked on the 3D TME dissection plane (red arrow in Fig. 2a). Subsequently, a point from where the marked point on the right side of the anal canal could be accessed without any hindrance was marked (blue arrow in Fig. 2a). By extrapolating the line connecting these two points to the abdominal surface and repeating this procedure (Fig. 2b), we determined the right boundary on the abdominal surface from where the marked point on the right side of the anal canal can be accessed without any hindrance (green arrows and green line in Fig. 2b). As shown in Fig. 2c, the maximum distance from the navel to the right boundary (A) and the distance from the navel to the right edge of the abdominal wall (B) were measured. The TME difficulty index was then defined as the ratio of these two distances (A/B) based on the assumption that the smaller the index, the greater the difficulty of accessing the deep pelvis and, consequently, the greater the difficulty of TME.

### MRI pelvimetry

**Figure 3:**
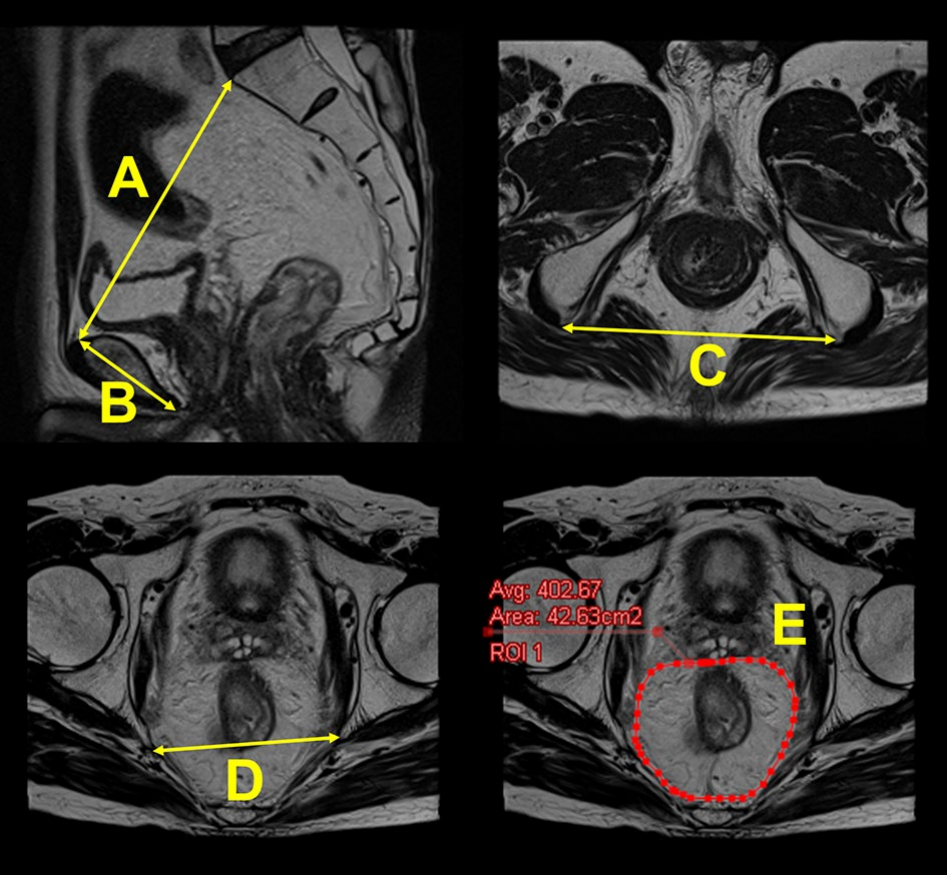
Magnetic resonance imaging pelvimetry. (A) Pelvic inlet (distance between the sacral promontory and superior aspect of the pubic symphysis). (B) Pubic tubercle height (distance from the superior to the inferior middle aspect of the pubic symphysis). (C) Intertuberous distance (distance between the lowest points of the ischial tuberosities). (D) Interspinous distance (distance between the tips of the ischial spines). (E) Mesorectal fat area at the level of the tips of the ischial spines (red circle).

The following five representative MRI pelvimetry parameters that were reported to be associated with the difficulty of rectal surgery in at least one study were measured using the T2-weighted MRI images (Fig. 3): interspinous distance (distance between the tips of the ischial spines), intertuberous distance (distance between the lowest points of the ischial tuberosities), pelvic inlet (distance between the sacral promontory and superior aspect of the pubic symphysis), pubic tubercle height (distance from the superior to the inferior middle aspect of the pubic symphysis), and mesorectal fat area at the level of the tips of the ischial spines^21^.

### Evaluation of surgical difficulty

In this study, we defined the operative time for TME as the time from the beginning to the end of TME dissection. The operative time for TME was determined by reviewing the operation video for each patient. We used the operative time for TME as an objective indicator reflecting the difficulty of TME. In addition to the analysis for all surgical types (LAR, ISR, and APR), we also conducted the analysis with the LAR cases only (*n* = 45) to minimize the bias caused by the different surgical types.

### Statistical analysis

All statistical analyses were performed using JMP version 12.2.0 (SAS Institute, Berkeley, CA, USA). Continuous variables were presented as the median (interquartile range), whereas categorical variables were presented as the number (percentage). Univariate regression analysis was conducted to evaluate the association between the operative time for TME and TME difficulty index or various clinicopathological and MRI pelvimetry parameters that were considered to be associated with surgical difficulty, including sex, body mass index (BMI), distance from the anal verge, tumor size, presence or absence of CRT, interspinous distance, intertuberous distance, pelvic inlet, pubic tubercle height, and mesorectal fat area. Variables with a *P* value < 0.05 in the univariate analysis were included in the multiple regression analysis. The standardized regression coefficient (*β* value) was used to estimate the effect of each explanatory variable on the objective variable. A *P* value of 0.05 was considered statistically significant.

## Results

### Clinical, surgical, and pathological features of the study participants

The clinical, surgical, and pathological features of the 66 patients with lower rectal cancer who underwent robot-assisted surgery are summarized in Table 1. The median age of the patients was 62 years, and their median BMI was 22.6 kg/m^2^; most were male (59.1%). The lower edge of the tumor was located at the rectum, below the peritoneal reflection and anal canal in 83.3% and 16.7% of the patients, respectively. The median distance from the anal verge to the lower edge of the tumor was 5 cm.

**Table 1:** Clinical, surgical, and pathological features of all patients (*n* = 66).

Age (years)^a^	62 (54–70)
Sex	
Male	39 (59.1%)
Female	27 (40.9%)
BMI (kg/m^2^)^a^	22.6 (20.7–24.6)
Tumor location (lower edge of tumor)	
Rectum below the peritoneal reflection	55 (83.3%)
Anal canal	11 (16.7%)
Distance from anal verge to lower edge of tumor (cm)^a^	5 (3–6)
Tumor size (mm)^a^	28 (20–36)
Preoperative therapy	
Chemoradiotherapy	41 (62.1%)
Radiotherapy	4 (6.1%)
None	21 (31.8%)
Pathological T stage	
pCR	4 (6.1%)
T1	15 (22.7%)
T2	23 (34.8%)
T3	24 (36.4%)
T4	0 (0.0%)
Pathological N stage	
N0	51 (77.3%)
N1–3	15 (22.7%)
M stage	
M0	62 (93.9%)
M1	4 (6.1%)
Radial margin positive	0 (0.0%)
Distal margin (mm)	23.5 (12–35)
Distal margin positive	0 (0.0%)
Surgical types	
LAR	46 (69.7%)
APR	12 (18.2%)
ISR	8 (12.1%)
Lateral pelvic lymph node dissection	14 (21.2%)
Diverting stoma in LAR and ISR	40/54 (74.1%)
Total operative time (min)^a^	403 (329–504)
Operative time for TME (min)^a^	117 (85–167)
Total blood loss (ml)^a^	60 (20–160)
Conversion to open surgery	1 (1.5%)
Postoperative complications (Clavien–Dindo grade II or above)	19 (28.8%)
Anastomotic leakage	0 (0.0%)

*BMI* body mass index, *pCR* pathologic complete response, *LAR* low anterior resection, *APR* abdominoperineal resection, *ISR* intersphincteric resection, *TME* total mesorectum excision.

^a^Values are median (interquartile range).

Preoperative RT or CRT was conducted in 68.2% of the patients. None of them had pT4 disease. LAR was the most common procedure used (69.7%), followed by APR (18.2%) and ISR (12.1%). LLND was performed in 21.2% of the patients. Pathological complete response after CRT was achieved in 4 patients. A total of 15 (22.7%) patients presented with lymph node metastasis, and 4 (6.1%) had distant metastases. The median DM was 23.5 (12–35) mm. No patients had positive DM or RM. The median total surgery time was 403 (329–504) min, and the median operative time for TME was 117 (85–167) min. The median amount of blood loss was 60 (20–160) mL. Postoperative complications with Clavien–Dindo grade II or above occurred in 19 patients (28.8%). No patients experienced anastomotic leakage. One LAR patient experienced conversion to open surgery and was excluded from the following analysis.

### Univariate and multivariate regression analyses in all patients

The measurement results of MRI pelvimetry and for the TME difficulty index are summarized in Table 2. First, we analyzed all 65 patients. Univariate analysis was performed to evaluate the associations between the operative time for TME and the various factors that could affect surgical difficulty. As summarized in Table 3, male sex, a higher BMI, and a larger mesorectal fat area demonstrated statistically significant positive associations, whereas the TME difficulty index demonstrated a statistically significant negative association. The TME difficulty index was found to be most strongly correlated with the operative time for TME (*β* = − 0.560, *P* < 0.0001). No significant association was detected between the operative time for TME and distance from the anal verge, tumor size, presence or absence of CRT, and pelvimetry measurements of the interspinous distance, intertuberous distance, pelvic inlet, and pubic tubercle height.

**Table 2:** Measurement results of MRI pelvimetry and for the TME difficulty index in all patients (*n* = 65).

Factors	Value
MRI pelvimetry data	
Interspinous distance (mm)^a^	104.4 (93.5–109.1)
Intertuberous distance (mm)^a^	126.1 (116.3–134.1)
Pelvic inlet (mm)^a^	122.0 (115.6–131.5)
Pubic tubercle height (mm)^a^	51.2 (49.6–53.6)
Mesorectal fat area (cm^2^)^a^	22.9 (18.1–31.5)
3D TME image data	
TME difficulty index^a^	0.536 (0.485–0.651)

*MRI* magnetic resonance imaging, *TME* total mesorectum excision.

^a^Values are median (interquartile range).

**Table 3:** Univariate and multivariate regression analyses for operative time for TME in all patients (*n* = 65).

Factors	Univariate analysis		Multivariate analysis	
	*β* value	*P* value	*β* value	*P* value
Sex (male vs. female)	0.438	0.0003	0.132	0.292
BMI	0.343	0.005	0.109	0.328
Distance from anal verge	0.140	0.266		
Tumor size	0.216	0.083		
RT or CRT (with vs. without)	0.028	0.826		
Interspinous distance	− 0.219	0.080		
Intertuberous distance	− 0.189	0.133		
Pelvic inlet	− 0.197	0.115		
Pelvic tubercle height	0.170	0.177		
Mesorectal fat area	0.385	0.002	0.223	0.045
TME difficulty index	− 0.560	< 0.0001	− 0.398	0.0025

*TME* total mesorectum excision, *BMI* body mass index, *RT* radiation therapy, *CRT* chemoradiotherapy.

Multiple regression analysis was conducted to predict the operative time for TME using significantly correlated indicators from the univariate regression analysis. Among said indicators were sex, BMI, pelvic inlet, mesorectal fat area, and TME difficulty index. As summarized in Table 3, the TME difficulty index (*β* = − 0.398, *P* = 0.0025) and mesorectal fat area (*β* = 0.223, *P* = 0.045) were independent predictors for the operative time for TME.

### Univariate and multivariate regression analyses in patients who underwent LAR

Next, to minimize the bias caused by the different surgical types, we analyzed only those patients who underwent LAR (*n* = 45). The characteristics of these patients are summarized in Supplementary Table S1. As summarized in Table 4, multivariate regression analysis demonstrated results similar to those obtained for all patients—that is, the TME difficulty index (*β* = − 0.350, *P* = 0.022) and mesorectal fat area (*β* = 0.342, *P* = 0.015) were independent predictors for the operative time for TME.

**Table 4:** Univariate and multivariate regression analyses for operative time for TME in patients who underwent LAR (n = 45).

Factors	Univariate analysis		Multivariate analysis	
	*β* value	*P* value	*β* value	*P* value
Sex (male vs. female)	0.338	0.023	− 0.029	0.843
BMI	0.271	0.071		
Distance from anal verge	0.021	0.893		
Tumor size	0.426	0.004	0.209	0.125
RT or CRT (with vs. without)	− 0.018	0.909		
Interspinous distance	− 0.169	0.268		
Intertuberous distance	− 0.207	0.173		
Pelvic inlet	− 0.217	0.152		
Pelvic tubercle height	0.055	0.719		
Mesorectal fat area	0.529	0.0002	0.342	0.015
TME difficulty index	− 0.487	0.0007	− 0.350	0.022

*TME* total mesorectum excision, *LAR* low anterior resection, *BMI* body mass index, *RT* radiation therapy, *CRT* chemoradiotherapy.

## Discussion

To the best of our knowledge, this is the first study that established a method for 3D visualization of the TME dissection plane along with various anatomical structures and tumor factors. Furthermore, utilizing the created 3D images, we developed the TME difficulty index, which reflects accessibility to the deep pelvis. According to the regression analyses, compared with other correlated indicators, the TME difficulty index was the strongest predictor for the operative time for TME. The analysis limited to LAR cases demonstrated the same result.

Contrary to previous studies that reported the usefulness of MRI or CT pelvimetry for predicting the difficulty of rectal cancer surgery^21^, this study found that interspinous distance, intertuberous distance, pelvic inlet, and pubic tubercle height were not significant predictors of surgical difficulty. These bone-based measures have the advantage of being easily measurable and highly reproducible; however, their greatest limitation is that they are not measured based on the actual TME dissection plane. Even in patients with the same pelvic skeletal structure, the distribution of soft tissue, including fat, muscle, and blood vessels, varies per patient. This may explain why the bone-based measures showed inferior ability for predicting surgical difficulty in our cohort relative to the soft tissue-based measures, such as the TME difficulty index and mesorectal fat area.

Similar to this study, several previous studies reported the usefulness of soft tissue–based measures. Yamaoka et al.^10^ reported that a larger mesorectal fat area at the level of the tips of the ischial spines was significantly associated with a longer pelvic dissection time during robotic resection for rectal cancer. Escal et al.^11^ reported that a mesorectal fat area > 20.7 cm^2^ at the level of the tip of the fifth sacral vertebra was significantly associated with greater surgical difficulty in patients with rectal cancer who underwent laparoscopic, robotic, or open resection. Another study reported that the visceral fat area best predicted the difficulty of laparoscopic rectal surgery^18^.

Several studies reported a significant correlation between BMI, a simple index of the degree of obesity, and the difficulty of rectal cancer surgery^11,15^. In this study, BMI was not significantly correlated with the operative time for TME and that for transecting the rectum in LAR. BMI reflects the amount of total body fat. However, it can be affected by muscle and bone mass and is not a good indicator of local fat distribution, including that in the pelvic region. Moreover, most studies in Western countries have used a BMI of 30 kg/m^2^ as the cutoff value for obesity, whereas in this study, the median BMI was 22.2 kg/m^2^ and only three patients had a BMI > 30 kg/m^2^. Thus, the influence of BMI on surgical difficulty might be limited in this study. Furthermore, several studies from Asian countries have suggested the absence of a statistically significant association between BMI and difficulty of rectal cancer surgery^10,18^.

This study has some limitations. First, a universal method for evaluating surgical difficulty does not exist. Most previous studies have focused on total operative time and total blood loss; however, these parameters can be easily biased by the type of surgery, such as LAR, ISR, or APR, and procedures with or without lateral pelvic lymph node dissection. Other candidate parameters for evaluating surgical difficulty include conversion rate to open surgery, anastomotic leakage, and TME specimen quality, among others. However, both the conversion rate to open surgery (1.5%) and anastomotic leakage rate (0.0%) in this study were very low and not suitable as indicators of surgical difficulty. As TME specimen quality is not routinely assessed in our institution, we adopted the operative time for TME in evaluating surgical difficulty—because TME is conducted as the standard procedure in patients with lower rectal cancer, regardless of the type of surgery performed. Inconsistencies in the methods used for evaluating the difficulty of rectal cancer surgery need to be addressed.

Second, this is a retrospective study with a limited number of patients in a single cohort. Thus, studies using a larger sample size are required to confirm our findings. Third, as robot-assisted rectal surgery has become the first choice in our institution in recent years, this study included robotic surgery cases only. Further research is needed to determine whether our results can be applied to laparoscopic rectal surgery cases.

In conclusion, compared with conventional bone-based pelvimetry measures, soft tissue–based measures, such as the novel TME difficulty index herein described and mesorectal fat area, might be more useful in predicting the difficulty of rectal cancer surgery. The 3D visualization of the TME dissection plane and predictive indicators of surgical difficulty would help surgeons identify potentially difficult cases and design appropriate surgical plans for a complete TME.

## Supplementary Information

Supplementary Table S1.

## Data Availability

The datasets generated during the current study are not publicly available but are available from the corresponding author on reasonable request.
