# Supplementary material for: Three-dimensional visualization of the total mesorectal excision plane for dissection in rectal cancer surgery and its ability to predict surgical difficulty
Source: Sci Rep. 2023 Feb 6;13:2130. doi: 10.1038/s41598-023-29426-x (PMC9902389; doi:10.1038/s41598-023-29426-x)
Supplement: Supplementary file 1 — Supplementary Table S1. [file 41598_2023_29426_MOESM1_ESM.docx]

| **Supplementary Table S1** Clinical, surgical, and pathological features of patients who underwent LAR (*n* = 45) | |  |
| --- | --- | --- |
| Age (years)^1^ | 63 (55–70) | |
| Sex |  | |
| Male | 27 (60.0%) | |
| Female | 18 (40.0%) | |
| BMI (kg/m^2^)^1^ | 22.7 (21.1–24.3) | |
| Distance from anal verge to lower edge of tumor (cm)^1^ | 5 (4–7) | |
| Tumor size (mm)^1^ | 27 (18–35) | |
| Preoperative therapy |  | |
| Chemoradiotherapy | 29 (64.4%) | |
| Radiotherapy | 1 (2.2%) | |
| None | 15 (33.3%) | |
| Pathological T stage |  | |
| pCR | 2 (4.4%) | |
| T1 | 11 (24.4%) | |
| T2 | 16 (35.6%) | |
| T3 | 16 (35.6%) | |
| T4 | 0 (0.0%) | |
| Pathological N stage |  | |
| N0 | 34 (75.6%) | |
| N1–3 | 11 (24.4%) | |
| M stage |  | |
| M0 | 43 (95.6%) | |
| M1 | 2 (4.4%) | |
| Radial margin positive | 0 (0.0%) | |
| Distal margin (mm) | 20.0 (13–31) | |
| Distal margin positive | 0 (0.0%) | |
| Lateral pelvic lymph node dissection (LLND) | 10 (22.2%) | |
| Diverting stoma | 20 (44.4%) | |
| Total operative time (min)^1^ | 394 (326–484) | |
| Operative time for TME dissection (min)^1^ | 116 (85–173) | |
| Total blood loss (ml) |  | |
| Postoperative complications (Clavien–Dindo grade II or above) | 7 (15.6%) | |
| Anastomotic leakage | 0 (0.0%) | |
| LAR, low anterior resection; BMI, body mass index; APR, abdominoperineal resection; ISR, intersphincteric resection; pCR, pathologic complete response; LLND, lateral lymph node dissection; TME, total mesorectum excision.  ^1^Values are median (interquartile range). | |  |
